# Effects of Functional Strength Training Combined with Aerobic Training on Body Composition, Physical Fitness, and Movement Quality in Obese Adolescents

**DOI:** 10.3390/nu16101434

**Published:** 2024-05-09

**Authors:** Zhihai Wang, Huihui Ma, Weiwei Zhang, Yufeng Zhang, Layale Youssef, Marcelo A. S. Carneiro, Chao Chen, Dan Wang, Dexin Wang

**Affiliations:** 1 School of Athletic Performance, Shanghai University of Sport, Shanghai 200438, China; wzh1026sus@163.com (Z.W.); mahuihui3752@163.com (H.M.); chenchao2019@sus.edu.cn (C.C.); wangdan@sus.edu.cn (D.W.); 2 School of Physical Education, Anhui Normal University, Wuhu 241002, China; 18895372433@163.com; 3 School of Exercise and Health, Shanghai University of Sport, Shanghai 200438, China; 2021518006@sus.edu.cn; 4 École de Kinésiologie et des Sciences de l’Activité Physique, Université de Montréal, Montreal, QC H3T 1J4, Canada; layale.youssef@umontreal.ca; 5 Metabolism, Nutrition and Exercise Laboratory, Physical Education and Sport Center, Londrina State University, Londrina 86050-070, Paraná, Brazil; mrclgee@gmail.com; 6 Applied Physiology, Nutrition and Exercise Research Group (PhyNEr), Federal University of Triangulo Mineiro, Uberaba 38025-180, Minas Gerais, Brazil

**Keywords:** obesity, exercise, combined training, physical health

## Abstract

This study aimed to compare the effects of 12 weeks of functional strength training combined with aerobic training (TG) and traditional resistance training combined with aerobic training (CG) on the body composition, physical fitness, and movement quality of obese adolescents. Forty participants were randomly assigned to either the TG group (*n* = 20) or the CG group (*n* = 20). Each group underwent training five times per week, lasting 120 min each time, over a total period of 12 weeks. All participants followed a strict dietary program. Anthropometric parameters, body composition, physical fitness, and movement quality were evaluated at baseline and after intervention. A two-way repeated measures ANOVA observed a significant interaction between time and group for body mass (*p* = 0.043), body fat percentage (*p* = 0.045), body mass index (*p* = 0.025), neck circumference (*p* = 0.01), chest circumference (*p* = 0.027), left-hand grip strength (*p* = 0.043), right-hand grip strength (*p* = 0.048), standing broad jump (*p* = 0.044), and total Functional Movement Screen score (*p* = 0.003), and the improvement was greater for TG in comparison to CG. TG was found to be more effective than CG in enhancing body composition, physical fitness, and movement quality in obese adolescents.

## 1. Introduction

In recent years, the sustained increase in obesity rates among children and adolescents has become an undeniable fact. In 2022, the number of obese girls and boys in 200 countries worldwide was 65.1 million and 94.2 million, respectively, an increase of 51.2 million and 76.7 million, respectively, compared to 1990 [1]. It is estimated that by 2030, there may be 1.1 billion obese adolescents worldwide [2]. Adolescent obesity is mainly caused by the interaction of poor dietary habits, a sedentary lifestyle, and a lack of physical activity [3]. Alarmingly, obesity in adolescents not only leads to unhealthy body composition but also significantly elevates the risk of severe psychological issues such as low self-esteem and depression [4]. Compared to their normal-weight counterparts (18.5–24.9 kg/m^2^) [5], obese adolescents display inferior physical fitness levels, including balance, strength, and speed [6,7,8]. Furthermore, they tend to have poorer movement quality, and obesity itself can contribute to these changes in movement quality [9,10]. The decline in physical fitness and movement quality in obese adolescents directly leads to an increased risk of injury, which in turn limits their engagement in physical activities [10,11,12,13]. Adolescence has been identified as a critical period for the onset and persistence of obesity [14], as well as a key window for improving health through exercise training [15]. Without a timely and effective intervention, up to 80% of adolescents will remain obese in adulthood, which can lead to elevated morbidity and mortality rates from conditions such as cardiovascular diseases, diabetes, hypertension, and various types of cancer [16,17]. Therefore, implementing interventions aimed at weight loss, physical fitness enhancement, and movement quality improvement is crucial for obese adolescents, both in the short and long term.

Public health guidelines recommend traditional strength training with aerobic training (CG; participants use weights, weight machines, and resistance bands to increase their ability to exert or resist force while also completing aerobic exercises (such as running and cycling) that involve numerous muscle groups [18]) as a non-pharmacological approach to improve body composition (body fat percentage, waist circumference, and body mass, etc.), physical fitness (muscle strength, speed, etc.), and movement quality in obese adolescents [19,20,21,22]. Numerous studies have demonstrated the superiority of CG over single exercise modes such as traditional strength training (TST) or aerobic training alone in improving body composition and physical fitness [15,19,23,24,25]. Combined training might have additive effects through greater volume of exercise or a combination of aerobic training (improvement in the oxidative metabolism–dependent energy system, fiber types, and metabolic capacity in skeletal muscle) and resistance training (quantitative changes in skeletal muscle mass or fiber diameter and increased muscular strength) [19]. However, in recent years, functional strength training (FST) has become a highly attractive training method and has garnered more interest in the sport science field. FST originated in the field of rehabilitation and physical therapy [26]. Compared with TST, this method is characterized by its multi-joint, multi-plane approach, providing comprehensive training that engages all muscles across all joints in the chain of functional movement. It emphasizes the holistic development of the body’s movement system [27] and has demonstrated its effectiveness in various populations. Studies have reported that FST can effectively improve the body mass, body mass index (BMI), and body fat percentage of middle-aged and elderly individuals [28,29,30] as well as upper body strength, lower extremity explosiveness, speed ability, and balance in various populations [26,27,29]. Additionally, FST can also effectively enhance movement quality, thereby improving physical fitness and reducing injury risk in adolescents and adults [29,31,32,33]. While the benefits of CG on body composition and physical fitness are well established, FST may offer advantages over TST, particularly in terms of movement quality. However, it remains unclear whether a combination of functional strength training and aerobic training (TG) is more effective than CG in enhancing body composition, physical fitness, and movement quality among obese adolescents.

Thus, the purpose of this study was to analyze and compare the effects of TG and CG on body composition, physical fitness, and movement quality in obese adolescents.

## 2. Materials and Methods

### 2.1. Participants

A total of 40 obese adolescents (24 males, 16 females) with simple obesity were recruited from the Guangzhou Sport and Weight Loss Summer Camp 2021. They were diagnosed with an abnormal accumulation of fat without any underlying disease [3] by general practitioners from the First People’s Hospital of Huizhou. A sample size calculation (G*Power 3.1.9.2, Franz-Faul, Universität Kiel, Germany, α = 0.05, power = 0.80, and effect size of 0.53 [34]) revealed a minimum of 31 participants would be required in our study. The participants’ body fat percentage was between 30 and 39.9 kg/m^2^ (within the range of mild and moderate obesity) [5]. Separately, they reported a low physical activity level (International Physical Activity Questionnaire [35]). They were randomly assigned to either TG (*n* = 20) or CG (*n* = 20). The characteristics of the participants are presented in Table 1. The inclusion criteria were as follows [36]: (1) adolescents aged 12–15 years with simple obesity (obesity was defined according to the criteria of the World Health Organization [5]); (2) a doctor’s recommendation for participation in the present experiment; (3) good compliance and the ability to adhere to the completion of the test and the training program; and (4) no history of surgery in the last three months. Exclusion criteria [36]: (1) secondary obesity and drug-induced obesity; (2) various cardiovascular diseases, diabetes mellitus, and dyslipidemia; (3) chronic alcoholics, smokers, and medication users; (4) regular exercise (more than two times per week, more than 20 min per session) within three months before the experiment; (5) contraindications to exercise; (6) unwillingness to participate in exercise at a scheduled time and place; (7) presence of a serious cognitive deficit that could prevent them from following instructions correctly; and (8) unwillingness of participants or guardians to sign an informed consent form. All participants and their parents signed informed consent forms prior to this experiment. This study was approved by the Human Research Ethics Committee of Shanghai University of Sport (No. 102772023RT205).

### 2.2. Study Design

**Figure 1:**
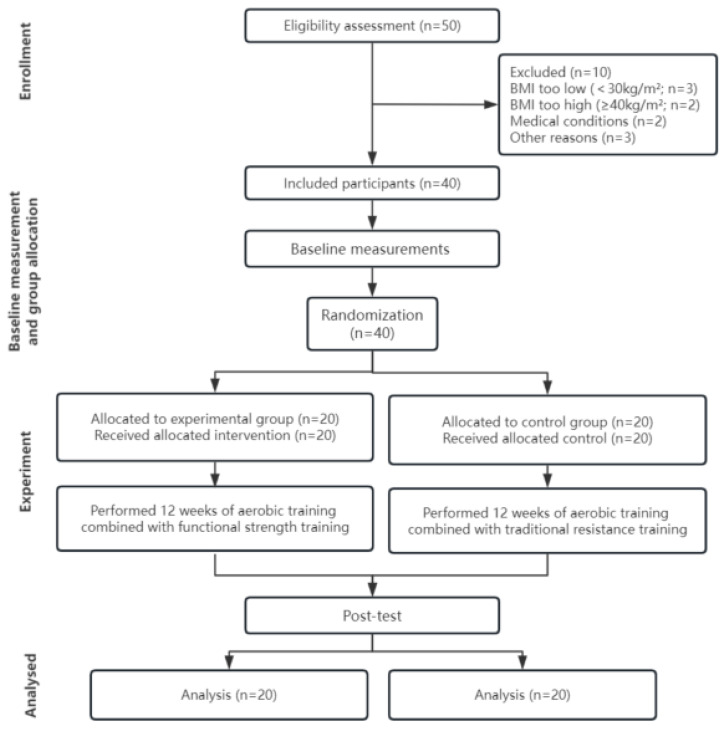
Flowchart of this experiment.

This study employed a randomized controlled trial design, with TG and CG undergoing assessments at baseline and after a 12-week intervention period (48 h following the final training session). The assessments included the following: (1) anthropometric and body composition measurements, such as height, weight, body fat percentage, BMI, neck circumference (NC), chest circumference (CC), waist circumference (WC), hip circumference (HC), and waist-to-hip ratio (WHR); (2) physical fitness tests, comprising flexibility (sit-and-reach), lower body strength (standing broad jump), upper body strength (handgrip strength), speed (50 m sprint), and (3) movement quality (Functional Movement Screening, FMS). Each measurement was taken at the same time of day (within a 1 h window) and under identical environmental conditions. A flowchart detailing this study’s procedure is presented in Figure 1.

### 2.3. Training Protocols

The sport and weight loss camp implemented a uniform closed management system which included consistent exercise and dietary regimens. Prior to the commencement of the experimental training, each participant received a detailed training plan. Under the guidance and following the demonstration of a strength and conditioning coach, all participants became familiarized with the training program and safety measures before engaging in the experimental training. Both TG and CG underwent 60 training sessions spanning over 12 weeks, at a frequency of five days per week. Each training session lasted 120 min and consisted of a 10 min warm-up involving jogging and dynamic stretching exercises, followed by 50 min of strength training, a subsequent 40 min of aerobic training, and a 10 min rest period between the two types of training. The training session concluded with a 10 min relaxation phase that incorporated static stretching exercises. The strength training programs for TG and CG were FST and TST, respectively. Throughout the exercise sessions, multiple strength and conditioning coaches were present to provide guidance and supervision, maintaining an instructor-to-trainee ratio of 1:10.

### 2.4. Aerobic Training

Aerobic training was primarily conducted using treadmills, bicycles, and elliptical machines. Heart rate monitors (Polar Electro Oy, Kempele, Finland) were used during exercises to monitor and regulate participant heart rate. The intensity of aerobic training was controlled at 65% HRmax [15].

### 2.5. TST Program

**Figure 2:**
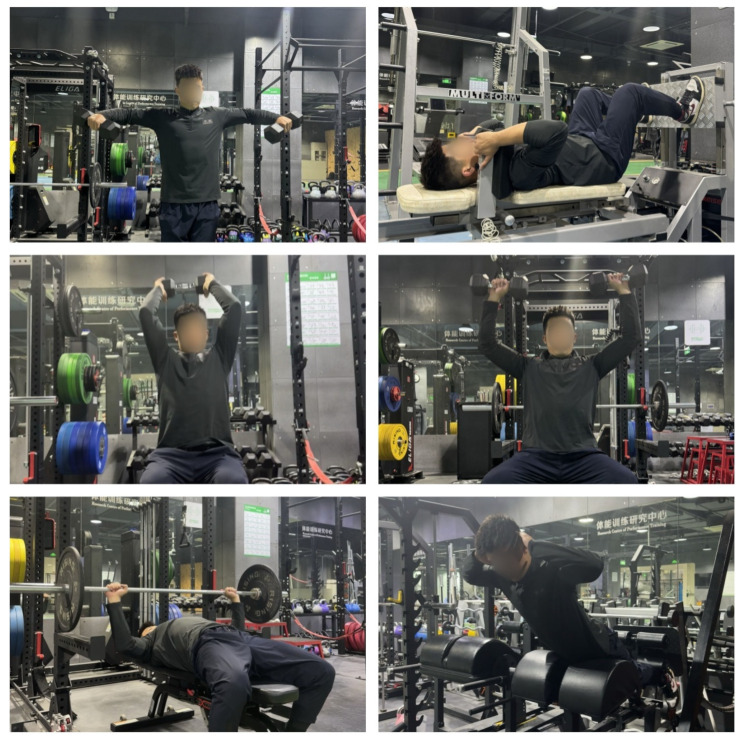
Example of traditional strength training.

The TST program primarily involved fixed-equipment exercises and self-weight training, targeting three main body regions: upper limbs, lower limbs, and the trunk. The program emphasized isolated muscle training, including exercises such as: pectoralis major (barbell bench press), biceps (bicep curls), rectus abdominis (sit-up), latissimus dorsi (seated pull-down), and quadriceps (seated leg press). During the initial two weeks (familiarization phase), participants engaged in self-weighted exercises such as kneeling push-ups, inclined supine back-presses, standing elastic band raises, supine curls, supine back raises, supine tucked leg raises, self-weighted deep squats, self-weighted cross-legged lunges, and self-weighted diagonal step squats. In weeks 3–12, participants cycled through five different groups (A, B, C, D, and E), performing 8–12 repetitions of each exercise in 2 sets. They rested for 2–3 min between sets and an exercise intensity of 60% -70% one-repetition maximum (1RM) (7–10RM predicts 1RM). Table 2 and Figure 2 provide a detailed overview of the training program along with visual representations.

### 2.6. FST Program

The FST program primarily involved synchronized, multidimensional, and multiple-joint movement modes that engaged multiple joints and muscle groups within the functional movement chain [27]. Given that the participants were obese adolescents without a foundation in exercise training, the FST training program comprised three different training phases. Exercises in the adaptation and familiarization phase (1–2 weeks) included four-point prone support, six-point prone support, supine support (shoulder and foot), supine support (elbow and foot), unilateral straight leg lower, kneel (one leg), kneel (both legs), basic posture of movement (both legs), basic posture of movement (one leg), straight leg hamstring stretch, wall squat, shoulder, blades rotation, Bosu ball stand, bridge, bridge–dog, overhead squat, alternating lunge, plank, and side plank. Exercises in the improvement phase (3–6 weeks) included banded overhead squat, overhead squat with band, wrist banded push-ups, trunk rotation (dumbbells), banded bird-dog, single-leg squat, incline body rows, incline push-up or kneeling push-up, multidirectional lunge with two dumbbells, kettlebell deadlift, Swiss ball-supported squat, kettlebell lumberjack, medicine ball push-up, medicine ball rotation throws, Swiss ball sitting balance, and knee tuck on stability ball. Health-related FST (7–12 weeks) exercises included one-leg Bosu ball stand, one-handed dumbbell/kettlebell pull, squat jumps, stability ball push-up, drop jump, modified Turkish get-up, forward and backwards medicine ball throw, landmine clean and press, medicine ball push-up in supine position, medicine ball release on the ground, medicine ball throw on the wall, stability ball chest press, single- or double-arm kettlebell swing, kettlebell sumo squat, medicine ball burpees, plank with Swiss ball, and side plank with Swiss ball.

**Figure 3:**
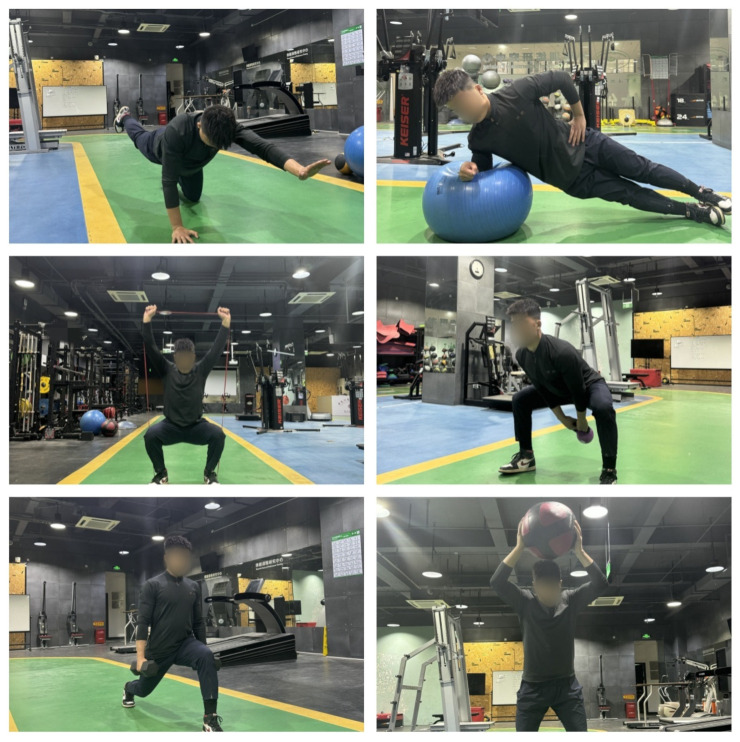
Example of functional strength training.

Exercise intensity was monitored using the rate of perceived exertion (Borg CR-10) (RPE), i.e., a fatigue rating (10) of 6–7 [37]. To ensure a consistent level of intensity throughout the 12-week intervention, self-weighted exercises were performed with an incremental increase of 10% in the total number of repetitions or total time. Meanwhile, free-weight exercises were performed increasing the total weight in the upper extremity by 5% and the total weight in the lower extremity by 10% per week [38]. A partial visualization is shown in Figure 3.

### 2.7. Diet Program

To mitigate the impact of dietary habits on study outcomes, both groups received identical diets during the summer camp and abstained from any nutritional supplements throughout the intervention period. The basal metabolic rate (BMR) was calculated using the Mifflin formula [39] (Men_BMR_ = 10 × weight (kg) + 6.25 × height (cm) − 5 × age (y) + 5; Women_BMR_ = 10 × weight (kg) + 6.25 × height (cm) − 5 × age (y) − 161), which considers the body mass and age of obese adolescents. Based on their basal metabolic rate, the adolescents were assigned to different groups, and a dietitian composed a personalized meal plan that considered their preferences and the Chinese Food Composition Table [40]. Specialized staff distributed each meal strictly according to the prescribed plan, and meal records were maintained. The dietary structure consisted of 50–55% carbohydrates, 30% lipids, and 15–20% proteins, while the energy distribution for the three meals was approximately 3:4:3 [41].

### 2.8. Anthropometric and Body Composition Assessments

Anthropometric and body composition indicators mainly included height, body mass, body fat percentage, BMI, NC, CC, WC, HC, and WHR. Height (to the nearest 0.1 cm) and body mass (to the nearest 0.1 kg) were measured using an electronic height and weight meter (Kaiyuan Electronics HW-600, Zhengzhou, China), and BMI was calculated using the formula (body mass/height^2^) [13]. Body fat percentage was measured using a body composition tester (Biospce InBody-370, Shanghai, China). Body circumference measurements were taken using the tape measure method for all circumferences, with an accuracy of 0.1 cm [36]. The neck was relaxed and the circumference between the upper edge of the subject’s 7th cervical vertebra and below the laryngeal node was measured as NC. To test WC, participants naturally stood with their arms crossed over their chest and feet together, and the mid-distance between the lower edge of the rib cage and the upper edge of the ilium was recorded. The horizontal circumference of the most prominent gluteus maximus muscle was taken as HC. The ratio of WC to HC is WHR. CC refers to the length of the tape measure around the nipples on both sides when the human body is in a supine position. To keep a certain consistency of the test procedure, all measurements were performed by the same person.

### 2.9. Physical Fitness Assessments

The primary physical fitness indicators assessed included flexibility, lower body strength, upper body strength, and speed, which are widely recognized measures for evaluating the physical fitness of Chinese adolescents [6]. Flexibility was evaluated using a Sit-and-reach tester (HK-6000, Hengkang Jiaye Technology Co., LTD., Shenzhen, China). Lower body strength was measured with a standing broad jump. Upper body strength was assessed using a grip dynamometer (WCS-110, Wanqing Electronics Co., LTD, Shanghai, China) with a precision of 0.1 kg. Speed was determined in a 50 m sprint timed with a Tianfu stopwatch timer (Fuhai Chemical Glass Co., LTD., Shenzhen, China). For all tests, the best result out of three attempts was reported and included in the final analysis, with a 3 min interval between each test.

### 2.10. Movement Quality Assessments

The FMS test developed by Gray COOK et al. [42] was utilized to evaluate the movement quality of the adolescents. The FMS test primarily examines adverse movement patterns that stem from poor stability, symmetry, and flexibility during human movement. The assessment protocol comprises seven distinct patterns: deep squat, in-line lunge, hurdle step, active straight leg raises, shoulder mobility, trunk stability push-up, and rotational stability. The three patterns, deep squat, lunge, and hurdle step, evaluate the three-foot positions of the body during movement (bilateral jumping, change of direction, and running). Active straight leg raises and shoulder mobility test are employed to assess the body’s primitive mobility patterns. Trunk stability push-ups and rotational stability tests focus on assessing the body’s core stability on both the coronal and sagittal planes. Each movement is categorized into four levels based on scoring criteria (0–3 score) [42]. The participants were evaluated according to each of the seven movement patterns by two experienced experts who used established scoring criteria [42]. Each pattern was assessed three times, with the highest scores being included in further data analysis. The cumulative score from each test was totaled to produce an overall FMS score, with higher scores indicating superior functional movement quality. The test procedure was recorded using video cameras positioned both in front and to the side. Furthermore, the participants underwent three supplementary exclusionary tests (shoulder impingement, trunk extension, and flexion tests) as per the screening guidelines to rule out any potential shoulder or back pain. However, since no participants reported experiencing pain during this study, these data were not included in subsequent analyses.

### 2.11. Statistical Analysis

Statistical analyses were conducted using SPSS, version 26.0 (SPSS Inc., Chicago, IL, USA). Data for the analyzed variables are presented as means and standard deviations. Data distribution was verified using the Shapiro–Wilk test, which showed that all the studied variables had an appropriate normal distribution. Two-way repeated measures analysis of variance (ANOVA) was used for data analysis [2 time-points (pre- vs. post-) × 2 group (TG vs. CG)]. The sphericity of the data was validated by Mauchly’s W statistic. Additionally, effect size (ES) was calculated using Cohen’s *d* [43] (the ranges used were the following: small < 0.06, moderate < 0.14, and large ≥ 0.14). The level of significance was set at 95% confidence interval (CI), and *p* < 0.05 was considered statistically significant.

## 3. Results

There were no statistical differences between EG and CG for any of the variables at baseline.

Forty participants completed the intervention program and reported no adverse effects. The percentages of adherence for both the TG and CG programs were 100%.

### 3.1. Anthropometric and Body Composition Results

**Figure 4:**
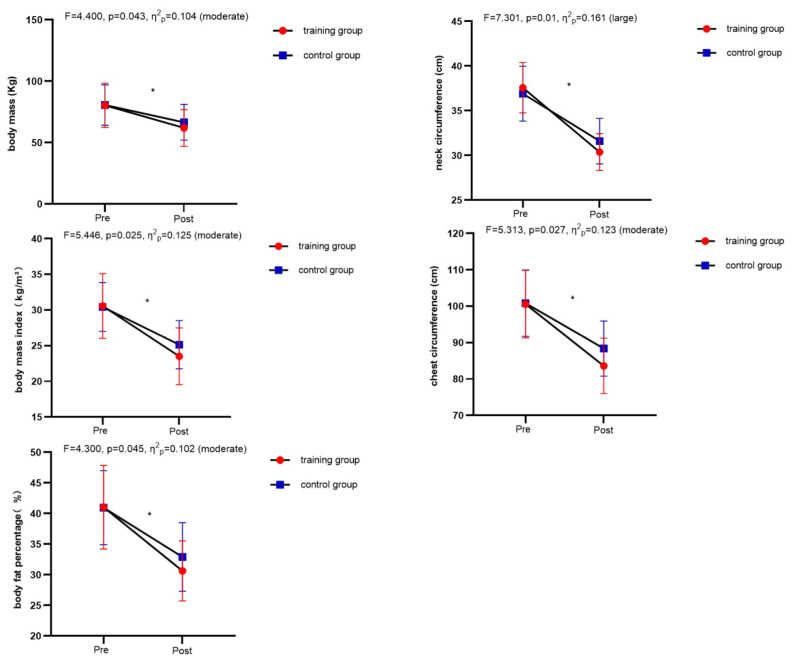
Comparison of anthropometric and body composition indicators between the training group and the control group before and after the intervention. * Significant difference from pre-test within the group.

Regarding anthropometric and body composition results, significant interactions between time and group were observed for body mass (*F* = 4.400, *p* = 0.043, *η*^2^*_p_*= 0.104), body fat percentage (*F* = 4.300, *p* = 0.045, *η*^2^*_p_*= 0.102), BMI (*F* = 5.446, *p* = 0.025, *η*^2^*_p_*= 0.125), NC (*F* = 7.301, *p* = 0.01, *η*^2^*_p_*= 0.161), and CC (*F* = 5.313, *p* = 0.027, *η*^2^*_p_*= 0.123; Figure 4). The main effect of time on WC [*F*(1,38) = 174.636, *p* < 0.01, *η*^2^*_p_*= 0.821], HC [*F*(1,38) = 147.165, *p* < 0.01, *η*^2^*_p_*= 0.795], and WHR [*F*(1,38) = 17.039, *p* < 0.01, *η*^2^*_p_*= 0.310] was significant, indicating both groups improved in terms of WC, HC, and WHR (*p* < 0.05).

### 3.2. Physical Fitness

**Figure 5:**
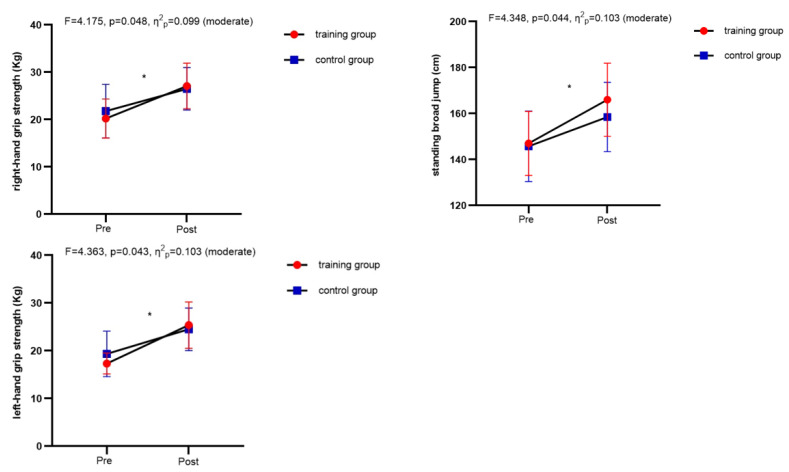
Comparison of physical fitness indicators between the training group and the control group before and after the intervention. * Significant difference from pre-test within the group.

Regarding physical fitness assessments, significant interactions between time and group were observed for left-hand grip strength (*F* = 4.363, *p* = 0.043, *η*^2^*_p_* = 0.103), right-hand grip strength (*F* = 4.175, *p* = 0.048, *η*^2^*_p_* = 0.099), and standing broad jump (*F* = 4.348, *p* = 0.044, *η*^2^*_p_* = 0.103; Figure 5). The main effect of time on speed [*F*(1,38) = 99.014, *p* < 0.01, *η*^2^*_p_* = 0.723] and flexibility [*F*(1,38) = 81.493, *p* < 0.01, *η*^2^*_p_* = 0.682] was significant, indicating both groups improved in terms of speed and flexibility (*p* < 0.05).

### 3.3. Movement Quality

**Figure 6:**
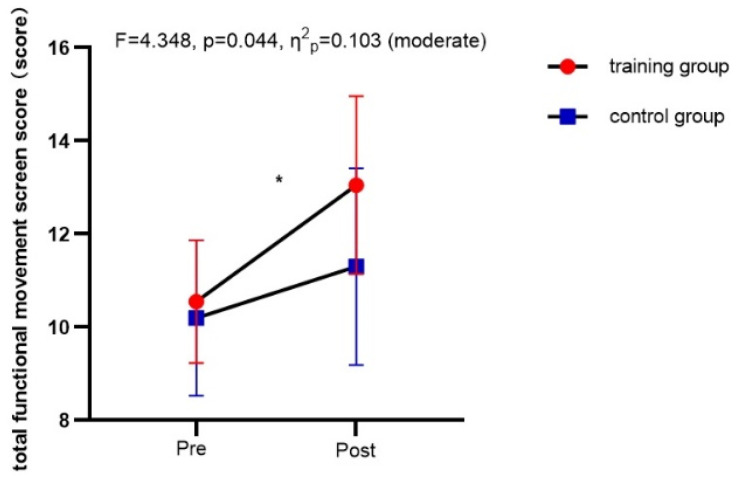
Comparison of movement quality indicators between the training group and the control group before and after the intervention. * Significant difference from pre-test within the group.

Regarding movement quality assessments, only total FMS scores (*F* = 9.698, *p* = 0.003, *η*^2^*_p_* = 0.203) were significantly affected by the interaction between time and group (Figure 6). The main effect of time on trunk rotational stability was not significant (*p* = 0.109). The main effects of time on deep squat [*F*(1,38) = 17.402, *p* < 0.01, *η*^2^*_p_* = 0.314], hurdle step [*F*(1,38) = 19.699, *p* < 0.01, *η*^2^*_p_* = 0.341], in-line lunge [*F*(1,38) = 11.400, *p* = 0.002, *η*^2^*_p_* = 0.231], shoulder mobility [*F*(1,38) = 12.761, *p* = 0.001, *η*^2^*_p_* = 0.251], active straight leg raises [*F*(1,38) = 6.840, *p* = 0.013, *η*^2^*_p_* = 0.153], and trunk stability push-up [*F*(1,38) = 7.435, *p* = 0.010, *η*^2^*_p_* = 0.164] results were all significant. There were no significant main effects of group on trunk rotational stability, deep squat, hurdle step, in-line lunge, shoulder mobility, active straight leg raises, or trunk stability push-up results (*p* > 0.05).

## 4. Discussion

The purpose of this study was to analyze and compare the effects of TG and CG on body composition, physical fitness, and movement quality indicators in obese adolescents. We hypothesized that both interventions would yield beneficial effects, with TG likely producing more significant improvements. The results partially support our research hypothesis that compared to CG, TG may be more effective in enhancing anthropometric and body composition (body mass, body fat percentage, BMI, NC, and CC), physical fitness (lower body strength and upper body strength), and movement quality (total FMS score) in obese adolescents. To the best of our knowledge, this study is the first to explore the effect of TG on obese adolescents. The findings of this study suggest that TG can be an effective method for improving body composition, physical fitness, and movement quality in obese adolescents.

Under unified dietary control, after 12 weeks of training intervention, obese adolescents in TG demonstrated more significant improvements in body mass, body fat percentage, BMI, NC, and CC compared to CG. As is widely recognized, FST elicits greater muscle activation than TST due to its dynamic and unstable nature. Moreover, heightened muscle contractions result in increased energy expenditure, fostering fat breakdown, the down-regulation of fat synthesis enzymes, and the suppression of fat production, thus enhancing overall body composition [29]. These findings confirm the results of previous studies. Ozkan et al. [29] found that 8 weeks of FST was more effective than TST in improving body mass and body fat percentage in middle-aged people. Meanwhile, Resende-Neto et al. [30] compared the effects of 8 weeks of FST and TST on the body composition of elderly women, pointing out that FST was more effective than TST in reducing fat mass. In addition, it was demonstrated that six months of FST (twice a week) could also effectively improve the body mass and BMI of elderly women [28]. The results obtained after the intervention showed that although there were no significant differences between the two interventions in terms of improving the WC, HC, and WHR of obese adolescents, both interventions induced some improvement in these parameters. It is important to recognize that both lower and higher BMI values may be connected with lower physical fitness [44]. A study by Matłosz et al. [44] found that among 13–19-year-old boys who regularly participate in sports, boys aged 14–15 had BMI values and body weight ranges of 19.2 ± 3.2 kg/m^2^~20 ± 1.7 kg/m^2^ and 53.5 ± 14.3 kg~59.6 ± 7.4 kg, respectively. Furthermore, it was revealed that boys with the lowest or highest BMI values performed the worst in physical tests, including strength and endurance. Consequently, investigating the relationship between BMI values and physical fitness could be a new direction for future research. This finding would not only help us to better understand the healthy development patterns of adolescents, but also holds significant importance for formulating effective physical education and health intervention measures.

Following 12 weeks of intervention, the TG group exhibited significant enhancements in lower body strength and upper body strength compared to CG. Given that the participants in this study were aged between 12 and 15 years old, it is notable that much of the strength development during adolescence stems from improvements in neuromuscular adaptability. Training programs can effectively mobilize the coordinated control ability of different neurons in the central nervous system, integrating motor efficiency and neuromuscular training. This approach ultimately facilitates the enhancement of strength quality and performance [27]. Compared with the TST program, which mainly focused on single joints and muscle groups, the FST training program was designed based on the coordination of all the joints in the body (such as exercises using kettlebells and medicine balls), which can better integrate motor efficiency and neuromuscular training, mobilize trunk strength in the human chain system to stabilize force and power transmission speed and power, and directly act on the integrated output of neuromuscular system strength [27]. This study supports existing research indicating that FST can effectively enhance the performance of upper and lower body strength qualities. Liaoting et al. [26] compared the effects of 12 weeks of FST combined with regular physical education classes and regular physical education classes on the strength qualities of 13–15-year-old middle school students, indicating that FST can more effectively improve upper body strength qualities. At the same time, Lamberth et al. [45] also reported similar results, showing that after 6 weeks of FST program, the upper and lower body strength of golfers increased significantly compared to the control group. Resende-Neto et al. [30] reported that FST was more effective than TST in improving women’s upper and lower body strength. Another study also showed that compared with a TST program, a 12-week FST program could more effectively improve the long-jump performance of 12–13-year-old girls [27].

In addition, the improvement in muscle strength may also be related to the improvement in movement quality, body fat percentage, as well as BMI in obese adolescents. Low FMS scores indicate possible problems such as poor stability, limited joint mobility, and restricted movement control [46]. Body flexibility and stability are the basis of human movement and can greatly affect movement ability and skill level [47]. In TG, the total FMS score increased significantly after the intervention, with an increase in FMS score from 10.55 to 13.05 points, which may have helped to improve the flexibility and stability of adolescents and thus enhanced the effectiveness of strength training. Previous studies have shown a positive correlation between muscle strength and total FMS scores in obese children [9]. In addition, some studies indicate a strong inverse relationship between physical fitness and body fat percentage as well as BMI. This means that higher levels of body fat and BMI can hinder flexibility and result in reduced strength quality [48]. This study shows that while the upper and lower body strength of teenagers in the TG group improved, their body mass, body fat percentage, and BMI indicators also improved significantly, which to some extent supports the above viewpoint.

On the other hand, we found that there was no significant difference between the TG and CG programs in improving the flexibility and speed of obese adolescents. This finding is inconsistent with the results of Liaoting et al. [27], who found that FST could significantly improve the flexibility of pre-adolescent girls, more so than TST. Generally, strength training does not promote flexibility unless specific flexibility training programs are added to strength training [49]. Moreover, differences in the study population may also lead to inconsistent results. Compared with normal-weight adolescents, obese adolescents have better flexibility than normal children and adolescents, and short-term non-specialized flexibility training may not have a significant impact on them [50]. Speed is influenced by multiple factors, such as anthropometry, strength, coordination, and technique. The correct technique is key to improving short-distance running performance and plays a role in reducing ground contact time [51,52]. The speed of obese adolescents did not significantly improve, which may be related to the fact that the training programs for TG and CG did not involve specific sprint training.

The results of this study indicate that the total FMS score of TG (10.55 to 13.05 points) was significantly improved after intervention compared to CG, but there was no significant improvement in its seven individual tests. This suggests that TG training programs are more effective than CG in improving the motor quality of obese adolescents. These findings are consistent with previous research results. In a study comparing the effects of FST and TST training, Liaoting et al. [27] found that female students aged 12–13 without a training background had a more significant improvement in total FMS scores after 12 weeks of FST than TST. In addition, compared to normal physical education classes, a 12-week TST program improved the total FMS scores of middle school students more effectively [26]. Another study on adults found that middle-aged men had better FMS scores after an 8-week FST program than those in the TST group [29]. The tasks included in FMS require optimal flexibility, muscle strength, functional range of motion, whole-body postural control, and balance [46]. The FST program involves a large number of multi-joint, unstable, and core stability training exercises, which help improve the total score of FMS in obese adolescents. In contrast, since the CG training program only involves movements along the sagittal axis (unidirectional), it has a greater impact on the development of single muscle groups [29]. As a result, the effect of TST programs on the motor quality of obese adolescents is limited.

At the same time, improvements in body mass, body fat percentage, and BMI indicators among obese adolescents may constitute significant factors affecting the improvement in motor quality. A study comparing the differences in FMS performance between normal-weight and obese children aged 10–11 found that there was a significant negative correlation between total FMS scores and BMI, and that obese children had poorer motor quality performance [53]. This indicates that obesity in children can lead to functional limitations, thus affecting motor quality performance. Our research findings revealed that while obese adolescents improved their body mass, body fat percentage, and BMI, their total FMS score also improved significantly. In addition to a significant improvement in total FMS scores, there was no significant increase in scores for the seven individual FMS tests, including deep squat, in-line lunge, and hurdle step. This may be because the movement quality of obese adolescents is significantly lower than that of healthy adolescents, and the 12-week intervention period seems to have had little effect on the results of each individual test.

This study has several limitations that warrant consideration. Firstly, since the participants were all attendees of a weight loss summer camp, a single aerobic training group was not included in this study. Secondly, this study was conducted in a closed training setting, thus caution should be taken into consideration when generalizing the findings. Thirdly, the sample size of this study was small. Lastly, the discrepancy between the chronological and bone age of the participants was not assessed, nor was the potential influence of natural growth during the study period controlled. Likewise, the testosterone levels in male participants were unknown.

## 5. Conclusions

This study observed that TG was more effective than CG in improving the body composition, physical fitness, and movement quality of obese adolescents. Specifically, TG significantly improved body mass, body fat percentage, BMI, NC, CC, upper body strength, lower limb strength, and total FMS score. These findings have significant potential for refining exercise prescriptions and optimizing health-related training outcomes in obese adolescents. Therefore, we recommend incorporating the TG program into the summer camp curriculum as an effective exercise prescription for weight loss. In the future, it would be worthwhile to investigate changes in participants’ blood biomarkers before and after the intervention. Additionally, it may also be worthwhile to combine this exercise prescription with school physical education courses to explore its long-term efficiency for obese adolescents.

## Figures and Tables

**Table 1:** Characteristics of the participants.

	TG (*n* = 20)		CG (*n* = 20)	
	Females (*n* = 8)	Males (*n* = 12)	Females (*n* = 8)	Males (*n* = 12)
Age (years)	12.9 ± 2.0	13.6 ± 0.9	12.9 ± 2.0	13.6 ± 1.2
Height (m)	1.61 ± 0.08	1.61 ± 0.09	1.60 ± 0.07	1.63 ± 0.09
Body mass (kg)	76.5 ± 14.9	83.1 ± 19.9	76.1 ± 14.9	83.7 ± 17.3
BMI (kg/m^2^)	29.2 ± 3.7	31.5 ± 4.9	29.5 ± 3.4	31.0 ± 3.5

Values are reported as means ± standard deviations (SDs); BMI = body mass index; TG = functional strength training combined with aerobic training group; CG = traditional strength training combined with aerobic training group.

**Table 2:** Twelve-week traditional resistance strength training program.

A	B	C	D	E
Bench press	Incline bench press	Chest fly	Decline barbell bench press	Bench press
Seated row	Lateral pulldown	Seated row	Seated row	Hyperextension
Lateral raise	Shoulder press	Front raise	Shoulder press	Lateral raise
Alternate dumbbell curl	Preacher curl	Dumbbell triceps Extension	Concentration curls	Alternate dumbbell curls
Tricep press	Assisted tricep dips	Squat	Leg press	Seated leg press
Squat	Straight leg raises	Seated leg press	Leg extension	Seated leg curl
Standing calf raise	Seated leg press	Standing calf raise	Lying leg curl	Barbell calf raise
Sit-up	Broomstick twist	Sit-ups	Broomstick twist	Sit-ups

A, B, C, D, E = weekly training program.

## Data Availability

The raw data supporting the conclusions of this article will be made available by the authors on request.
